# Exploring perinatal loneliness as a key social determinant of perinatal mental ill health in the UK: findings from a multidisciplinary consensus statement exercise that mapped knowledge about measurement, prevalence, antecedents, impacts and interventions, and agreed future priorities for research, policy and practice

**DOI:** 10.1136/bmjopen-2024-085669

**Published:** 2025-05-31

**Authors:** Ruth Naughton-Doe, Rebecca Nowland, Jacqueline Kent-Marvick, Phoebe McKenna-Plumley, Emily Lovett, Thuy-Vy Nguyen, Lindsay Hunter, Nicola Wallis, Florence Gaughan, Katherine Hall, Corinna Colella, Suzanne Wilson, Katherine Adlington, Elizabeth Taylor Buck, Sarah Shemery, Naomi Finch, Catrin Noone

**Affiliations:** 1 Business & Society, University of York, York, UK; 2 School of Community Health and Midwifery, University of Central Lancashire, Preston, UK; 3 College of Nursing, University of Utah, Salt Lake City, Utah, USA; 4 Queen’s University Belfast, Belfast, UK; 5 Edge Hill University, Ormskirk, UK; 6 Durham University, Durham, UK; 7 Birmingham City University, Birmingham, UK; 8 University of Cambridge, Cambridge, UK; 9 Sheffield Hallam University, Sheffield, UK; 10 University of Bristol, Bristol, UK; 11 University of Central Lancashire, Preston, UK; 12 East London NHS Foundation Trust, London, UK; 13 University of Sheffield School of Health and Related Research, Sheffield, UK; 14 Social and Political Science, University of Edinburgh School of Arts Culture and Environment, Edinburgh, UK; 15 SPSW, Univ York, York, UK

**Keywords:** Postpartum Period, Pregnancy, MENTAL HEALTH, Primary Prevention, Pregnant Women

## Abstract

**Abstract:**

**Objectives:**

New parents are at increased risk of loneliness, which adversely affects parental and infant health and well-being and has been linked to an increased likelihood of parental mental illness. In the UK, perinatal mental illness is estimated to cost £8.1bn a year, predominantly due to lasting poor health and developmental consequences for children. The purpose of this consensus statement is to determine the state of this research field, highlighting key issues for researchers, policymakers and those responsible for perinatal mental health services and interventions. We will also highlight knowledge gaps to be addressed in future perinatal loneliness research.

**Design, setting and participants:**

The Parental Loneliness Research Group held six online meetings between October 2023 and May 2024, attended by academics and practitioners from the UK and USA. Attendees conducted a mapping exercise by appraising published, unpublished and ongoing perinatal loneliness research. The findings were shared with advisory groups, including parents with lived experience of loneliness. A consensus statement was then drafted, reflecting existing knowledge and gaps in the current evidence about the experience of perinatal loneliness in the first 1001 days.

**Results:**

A consensus about the definitions, measurement, prevalence, antecedents, impacts and interventions relevant to perinatal loneliness is outlined. Gaps in the literature are highlighted.

**Conclusions:**

Despite emerging research into perinatal loneliness, it is hard to determine prevalence due to limited analyses of national survey data. Recommendations for future research include secondary data analysis; prioritising equality, diversity and inclusion; reconsidering solutions to perinatal loneliness through a social justice lens; co-producing interventions, and rigorous evaluation of existing interventions.

**STRENGTHS AND LIMITATIONS OF THIS STUDY**

- The statement incorporates perspectives of multidisciplinary academics and a wide range of practitioners and people with lived experience.
- We synthesise current knowledge using published academic research, unpublished ongoing findings, policy documents and practitioner and lived experience.
- A consensus meeting methodology was used to develop our consensus statement, a practical method to provide a rapid state-of-the-art understanding.
- We recognise that consequently some voices and experiences may be missing from this statement.
- While we included all published reviews on perinatal loneliness conducted between 2021 and 2025 and further papers identified through the consensus process, we did not conduct new systematic searches for this consensus statement.

## Introduction

 Perinatal loneliness refers to the loneliness experienced by parents and prospective parents during pregnancy and up to 2 years postbirth, including for partners and those who have children via adoption, surrogacy and other means.^1^ According to the WHO’s Commission on Social Connection (2024–2026), finding solutions to loneliness is an international public health and policy priority due to its negative impact on physical and mental health and the well-being of communities and societies.^2^ Perinatal loneliness also has profound intergenerational adverse effects on the well-being of children.^3^ The global understanding that we urgently need to address loneliness more generally has been accompanied by an associated exponential growth of policy and research focus. However, persisting limitations in the perinatal loneliness evidence base are hampering efforts to overcome it. Between October 2023 and May 2024, a group of UK and US-based academic researchers and practitioners formed a working group to establish a consensus statement about the current evidence base and identify priorities for future research. Participants in advisory groups for the Coproducing Solutions for Perinatal Loneliness project led by the first author, including 10 parents with lived experience and 18 professionals, were consulted throughout.

## Background

Perinatal mental health in the UK is in crisis, with an estimated maternal prevalence of 25.8%.^4 5^ Suicide continues to be a leading cause of direct maternal death between 6 weeks and 12 months after birth, accounting for a staggering 39% of deaths in this period,^5 6^ and the rate is rising.^7^ An estimated 18% of fathers also experience perinatal mental illness, though fathers are not routinely screened.^8 9^ Perinatal mental illness is estimated to cost society £8.1bn per year, mostly related to the lasting impact on children.^4^ Thus, the first 1010 days, from conception to 2 years, are critical for parents and infants.^9^

Preventing perinatal mental health problems from developing or worsening has consequently been identified as a priority by the UK Government^10^ and professional organisations such as the Royal College of Midwives.^11^ The National Maternity Review: Better Births (2016)^12^ and the NHS England ‘Three-year delivery plan for maternity and neonatal services’^13^ emphasise the importance of access to perinatal mental health support. There is also recognition of the need to offer preventative approaches, rather than reactive interventions.^10^ One promising strategy to prevent perinatal mental health problems is to tackle loneliness. There is strong evidence to indicate that loneliness is a causal and exacerbating factor for poor perinatal mental health.^314^,^16^ Loneliness is also a known predictor of suicidal ideation and behaviour.^17^ In support of this argument, the UK Government Loneliness Strategy identified tackling loneliness experienced by new parents as a priority in 2022, because of its profound impacts on physical and mental well-being.^18^

This paper synthesises current practice knowledge and research into perinatal loneliness and highlights urgent priorities for future research. We outline existing knowledge relating to key definitions, epidemiology, prevalence, measures, antecedents, consequences and effective interventions.

## Methods

The Parental Loneliness Research Group developed a consensus statement through a series of online meetings between October 2023 and May 2024. We followed a consensus meeting approach^19^ previously used to develop a similar statement for older adults.^20^ The first meeting was attended by academics and practitioners in the UK and USA who had attended the first Perinatal Loneliness Annual Conference in October 2024. It included those working in social policy, sociology, social work, psychology, midwifery and psychiatry, a parent-infant psychotherapist and people delivering perinatal creative and cultural interventions. We identified and invited other experts to subsequent meetings, including the authors of any literature reviews on perinatal loneliness.

The lead author took notes at each meeting and produced a list of points to consider based on Prohaska’s approach.^20^ These were research methods used in perinatal loneliness research; conceptual definitions; measurement and prevalence of perinatal loneliness; experiences, influences and causes of perinatal loneliness; individual, structural and cultural drivers and risk factors; impacts of loneliness; barriers to overcoming loneliness; interventions for overcoming loneliness and intervention mechanisms. Authors RN-D and RN developed a spreadsheet of the above items for co-authors to map key information from their published and unpublished research. Practitioners in the group completed a spreadsheet to reflect on their experiences of supporting parents to supplement insights from published papers. We then used the contents of the spreadsheets to structure our online discussions. Through a process of co-writing on a shared online document and discussions in our meetings, we agreed on a consensus statement. A summary of our conclusions is presented below.

## Patient and public involvement statement

The Lived Experience Advisory Group for the Coproducing Solutions for Perinatal Loneliness Project was consulted by the lead author (RN-D) to hear their perspectives during seven online meetings between January 2023 and June 2024.^21^ 10 parents contributed, including four fathers, one young parent (under 25), two solo parents, one parent with a disability, two parents identifying as lesbian, gay, bisexual, transgender or queer (LGBTQ+), two Muslim parents, one migrant parent, two military parents, three Asian parents and one Black parent. Two parents had experience of Mother and Baby Units for their mental health following birth. The Practitioners’ Advisory Group for the same project was also consulted. This group of 18 included midwives, health visitors, peer support workers, people working in voluntary sector services and people working in services for fathers.

## Definitions and dimensions

Perinatal loneliness literature has drawn on general conceptual definitions of loneliness, as specific definitions of loneliness experienced in the perinatal period are lacking. Loneliness refers to a personal and subjective feeling that one’s social connections do not meet a person’s expectations of quantity or quality.^22^ Other academics refer to feelings of loneliness resulting from an absence of meaningful relationships,^23^ or worldly purpose.^24^ While loneliness and social isolation are often conflated, the two are distinct, with social isolation referring to the objective state of having limited social contacts.^23^ Consequently, one can be physically isolated and not lonely, or be lonely while having an abundance of social connections.^23^

A further distinction is that of social and emotional loneliness, which have both been identified in the perinatal period.^1^ Social loneliness is the subjective experience that occurs when a parent feels that they lack an engaging social network, and emotional loneliness is the perceived absence of close emotional ties.^25 26^ Although less commonly studied, a further type of loneliness relevant in the perinatal period is existential loneliness, where a parent experiences a feeling of fundamental disconnection from other people.^21 27 28^

Loneliness in the perinatal period may also be situational or transient^1 22 29^ in that it is experienced during developmental changes or crises such as moving places, or jobs, bereavement, or having a baby. Transient loneliness has been viewed as less concerning than persistent loneliness^22^ because chronicity of loneliness has been associated with negative health and well-being impacts. However, loneliness in the perinatal period warrants concern because the first 1001 days are critical for parental and child well-being, healthy parent-infant relationships and child development.^3 9^

## Measurement and prevalence

The UK has limited accessible data on the prevalence and distribution of perinatal mental illness and its known risk factors, including loneliness. A review of national data sources, including NHS Digital (now NHS England), Public Health England and other national Perinatal Mental Health resources, identified a perinatal mental health data ‘blind spot’.^30^ There were significant inadequacies in the utility of all identified data sources, making it impossible to provide information on the UK prevalence and variation by sociodemographic differences.^30^ Despite this, there are strong indications that the prevalence of loneliness is generally high in parenthood. An international scoping review of 108 papers focusing on parents of children aged 0–5 reported that between 32% and 42% of parents felt lonely, increasing to 70% for parents of children with disabilities or poor health.^1^ A UK-based survey of over 2000 parents reported that half of the respondents had felt lonely in the past year,^31^ with similar prevalence rates reflected in longitudinal cohort studies.^32^

Where loneliness data are available, they do not often distinguish between different types of loneliness, which may need different types of support. Individual studies have measured loneliness using validated tools such as the UCLA Loneliness Scale^33^ and the de Jong Gierveld Loneliness Scale.^34^ These scales are indirect measures of loneliness (ie, do not use the words loneliness/lonely) and are useful to understand loneliness among groups who are reluctant to admit to, or may not recognise themselves as, being lonely. Although potentially useful for the perinatal population, who may regard feelings of loneliness as a failure to be the parent they hoped to be or feel they should be,^12^,^1421^ they were not developed specifically for this group and may not accurately represent their experiences. The Japanese three-item UCLA Loneliness Scale has been validated in the perinatal population in Japan,^35^ but existing loneliness measures need to be examined for appropriateness and validated for use in the UK perinatal population. New measures may also need to be designed to specifically measure perinatal loneliness. This is because existing loneliness scales need development, as some have psychometric flaws and items lacking content/face validity.^36^

Future research should explore large-scale government panel surveys. Some national surveys such as the Understanding Society and the Community Life Survey include measures of loneliness, with the Understanding Society survey most useful as it tracks changes in parental status and loneliness over time.^37^ Analysis of this could help establish causal mechanisms, in particular whether having a baby is associated with a change in loneliness rather than a continuation from existing loneliness. Another useful resource could be through collating and analysing pre-existing data collected through the routine use of patient-reported outcome measures (PROMs) in perinatal mental health services (PMHS). PROMs include a direct question about loneliness and are completed at a minimum of two time points during a service user’s journey through services.^38^ This is potentially an untapped source of data about perinatal loneliness.

## Causes

To our knowledge, there is no study in the UK that has quantitatively explored causes (antecedents) of perinatal loneliness, including different types, and identified specific risk factors. Cross-sectional survey research in Japan has shown that increased levels of maternal perinatal loneliness are associated with social isolation and having smaller support networks, an unsupportive partner, few people to consult with about parenting, insecure attachment and few friends who are parents.^39^,^41^

There are a growing number of qualitative studies which shed light on potential causes of loneliness in the UK. Most focus on maternal experiences, though one paper explicitly focusses on fathers^42^ and one project report explored all parents’ experiences.^21^ Our mapping exercise of published and ongoing research studies examined the nuance relating to the experiences of loneliness in the UK.^13 14 15 21 29 43^,^53^ We derived three themes relating to the key antecedents: (1) reconfiguration of relationships, social networks and support; (2) cognitions about parenthood and identity and (3) feeling separated, excluded or different. These themes are summarised below and align with the proposition that emotional, social and existential loneliness are prevalent in the perinatal period.

### Reconfiguration of relationships, social and support networks

Loneliness in the perinatal period is often accompanied by the rearrangement of social networks. Friendship, work, family and romantic relationships change and can lead to social loneliness.^115 21 43 45^,^52^ There is also a discrepancy between how much support is expected and how much is received from one’s social networks.^14 15 43 46^ Qualitative changes to relationships with partner, family and friends, or lacking the right kind of support from health and care services, partner or family members,^1 15 21 45^ lead to emotional loneliness, as parents feel alone with the emotional burdens of caring.

### Cognitions about parenthood and identity

Loneliness is often experienced in the overwhelming or unexpected nature of parenting that leads to parents feeling isolated.^14^,^1621 43 48^ Motherhood is assumed to be intuitive, but for those for whom it is not, it leads to feelings of isolation.^54^ Like other studies highlighting the increase in loneliness during transitory periods^50 55^ and linked with existential loneliness, parents also experience a sense of loneliness that is associated with a feeling of disconnection from their previous sense of identity and self,^1 15 21 48 52^ due to shifts in priorities focused more on the child’s needs than one’s own.^48 56^ Finding parenting harder than expected and not fitting their own or their perception of society’s parenting ideal led to feelings of failure.^1 21 29^ Parents sometimes masked their difficult feelings due to stigma or shame, or fear of child removal.^21^

### Feeling separated/excluded/different

Perinatal loneliness has been linked to marginalisation and is thought to be more prevalent where there are intersectional inequalities.^43^ Loneliness more generally has been framed as a social justice issue owing to its unequal distribution throughout society, affecting marginalised and vulnerable communities.^57^ Our mapping exercise identified that parents who felt isolated, excluded or different may feel lonely, and this included financial, social or cultural exclusion, such as those who are experiencing racism, ageism, sexism or homophobia.^1 43 45 48^ People who have migrated and are refugees and people from black or ethnic minority groups often face the most and multiple disadvantages.^43 48^ Fathers may experience loneliness in the perinatal period due to the expectations to provide support and be the breadwinner, combined with a culture where men are not encouraged to share their feelings.^21 42 54^ Normative assumptions about family makeup and who ‘should’ become parents contribute to discrimination and exclusion and loneliness for young mothers,^45^ LGBTQ+ parents, solo parents and ‘non-traditional’ families.^21 58^

It will be important in future research to explore and refine the themes identified here and consider how they relate to the different domains of loneliness, both through secondary data analysis involving cross-sectional research and longitudinal panel studies to examine causal relationships and qualitative synthesis to provide nuanced understanding of relationships and lived experiences of specific cohorts that are most likely to experience perinatal loneliness.

## Impacts

The experience of perinatal loneliness has been found to have vast impacts on the health and well-being of parents directly but also via transgenerational influences on children.^3^ There is an emerging literature revealing links between perinatal loneliness and poor mental well-being which is focused on postnatal depression.^1 3 14 15 45^ In contrast to literature in other populations, there have been few studies that have examined physical health and well-being and associations with perinatal loneliness.^3^ This will be an important area for future research. As physical health impacts of loneliness have been found in other populations, it is possible that similar findings may be evident in loneliness in early parenthood.^3^

Furthermore, studies have reported concurrent associations between parent and child loneliness,^59^ particularly in relation to the mother and their children.^60^ However, there may be a gender effect in the transmission of loneliness from parent to child because one study found that fathers’ loneliness was predictive of their sons’ persistent loneliness and mothers’ loneliness predictive of their daughters’.^61^

In addition, parental loneliness has been linked to children’s developmental outcomes. Mothers’ loneliness has also been associated with their child’s poorer problem-solving skills,^62^ poor social competence, hostility and fear of negative evaluation^63^ and social anxiety in girls.^64^ Both mothers’ and fathers’ loneliness are associated with poorer peer-evaluated co-operating skills in girls.^59^

While there have been no examinations of the social and economic impacts of perinatal loneliness, it is likely that there are wider costs to society. These include productivity losses from work absences, unemployment due to ill health (physical and mental) and difficulties re-engaging with a previous role following disengagement or leave of absence. The estimated cost of general loneliness to employers is £2.5 billion annually.^65^ Given the evidence that perinatal loneliness is linked to increased use of healthcare services,^66 67^ there will be economic costs of perinatal loneliness in relation to healthcare utilisation. While there have been no economic evaluations of perinatal loneliness, loneliness in old age has been estimated to cost health and social care services more than £1700 per person over a 10-year period, with estimated costs to be in excess of £6000 per person for the most severely lonely.^68^ Importantly, loneliness has been linked to postnatal depression, which is estimated to cost society around £74 000 per case.^4^

## Interventions

There are a range of services and activities delivered by statutory and voluntary sector services available for parents in the UK that are thought to reduce loneliness. The UK Government’s Best Start for Life Policy^9^ set up ‘Family Hubs’ in 75 local authorities in England to deliver local, co-produced, joined-up support for parents of 0–18 s, including perinatal mental health support. An independent evaluation of the Family Hubs has been commissioned with findings due in 2025–2026. While this provision shows promise of supporting parent and child well-being, it is only available in certain parts of England, and funding is time limited, which creates sustainability challenges.^69 70^ While ‘Family Hubs’ are intended to provide a local base for parent support, there are varying models, some with support being more geographically spread and/or involving outreach work.^70^ Evaluating the loneliness impacts of the different models will be important in understanding what works in reducing perinatal loneliness.

One scoping review of parental loneliness found health visiting to be effective.^16^ However, there has been a 46% decrease in spending on Early Years Services, Midwifery and Health Visiting services since 2010.^71 72^ Ensuring continuity of care in maternity service provision, including health visiting, is important for supporting parents, but for many, this is not a reality.^14 21^

Antenatal classes are also widely considered to reduce loneliness through supporting new parents to build social networks. However, there are issues around equitable access to such programmes, consistency of delivery and the impact of transitioning classes to online platforms on social connectedness. A Cochrane review of the obstetric and psychosocial outcomes of antenatal educational programmes found a lack of proper evaluation and a need for more thorough trials to examine effectiveness.^73^ The literature on social outcomes of antenatal classes has not been synthesised, but there are several qualitative studies that report proximate outcomes including obtaining peer support,^74^ building friendships^75 76^ and reducing isolation.^74^,^76^ The social outcomes of antenatal classes, an intervention widely believed to develop social networks, have not been robustly evaluated.^73^

Peer support can also help reduce loneliness either when delivered by a professional or a volunteer,^14^,^1648^ although the synthetic nature of a formal support relationship can further isolate once the support ends.^48^ Parents also felt less lonely when connected in a group of similar peers where they felt safe to share and normalise their feelings.^1 4 14 16 21 45 46 48 50 51^ Interventions that parents liked included Creative Health Interventions, such as walking and nature-, art- or music-based interventions.^77 78^ These were often designed for the parent, were child-friendly and provided opportunities for self-care, fun and self-expression.^77 78^

In addition to health visiting and peer support, one review found other interventions that may be effective included a universally provided child development programme, interpersonal-skills training and short-term cognitive behavioural therapy.^15^ Another scoping review of interventions for perinatal loneliness, including 50 studies, developed a typology of six types of interventions for loneliness and proximate determinants.^48^ These were synthetic social support, shared-identity social-support groups, creative health approaches, playgroups, place-based and multidisciplinary support that worked with parents to overcome a range of barriers to connection and awareness campaigns. Five mechanisms commonly used within these interventions were (1) opportunities for social connection to similar others, (2) positive relationships with a professional or volunteer, (3) normalisation and acceptance of difficulties, (4) meaningful activities and (5) support to overcome barriers (including cultural and financial) to connection.^48^ These studies reported positive effects, though a meta-analysis of outcomes was not possible as studies used a wide range of different research designs and outcomes. Only six studies aimed to measure changes to an outcome measure of loneliness, of which only one was a randomised controlled trial.

Future intervention studies should build on this research and test the efficacy of different types of intervention and different mechanisms using robust methodologies. The scoping review of interventions for perinatal loneliness found that only one study was co-designed with a clear process of public involvement, very few considered ethnicity, only one recorded sexuality and only one recorded participants’ religion.^48^ Given the importance of intersectionality when understanding experiences of loneliness, equality, diversity and inclusion should be at the forefront of studies seeking to develop or test interventions.

Future research should also focus on evaluating support provided by the plethora of national and local voluntary sector organisations which include large charities such as the National Childbirth Trust that run antenatal and postnatal courses and social groups, and PANDAs (Pre and Postnatal Depression Advice and Support), which supports parents with perinatal mental illness. There is increasingly a private sector offer of classes such as music, baby sensory or baby yoga. Many activities list reducing isolation as an aim, but this is usually secondary to baby development. These classes are often not accessible for all as they usually involve a cost. Further, some participants in the Perinatal Loneliness Lived Experience Research Advisory Group explained a perception that some of these activities were designed for Western, educated and middle-class families and may not feel welcoming to many communities. It will be important to evaluate the reach and social outcomes of these approaches using robust methodologies, including realist approaches.

The Perinatal Loneliness Research Advisory Group and the authors of the consensus statement identified that many activities provided in the perinatal period do not meet parents’ social needs because they are designed primarily for the child (eg, baby classes including baby massage and baby sensory and perinatal education classes). Services often assume that parents needed support with their parenting or bonding, rather than opportunities to socialise with others. Sometimes parents felt judged and compared themselves to others, or to the ‘best practice’ presented in the activities or course materials. General group activities focused mostly on the baby may reduce objective isolation but often had the unintended consequence of exacerbating feelings of loneliness by not creating sufficient opportunities for parents to talk to each other. Baby-focussed activities also normalised prioritising child well-being over parent well-being rather than supporting the parent and baby dyad.

Parents in the Advisory Groups also reported wanting opportunities to contribute their skills rather than being framed as needing support. Volunteering is a valued intervention to reduce loneliness for older people,^79^ but the scoping review of interventions found no interventions offering this for new parents.^48^ One organisation in the UK (Blaze Trails) encourages new parents to volunteer through leading community walks for parents and babies, and internal evaluations report favourable social outcomes including feeling part of a community and feeling valued.^51^ Recognising new parents for their strengths and encouraging their contributions rather than framing them as in need of support may help to reduce existential loneliness. It would be useful for future research to explore the feasibility and outcomes of volunteering in the perinatal period.

## A way forward: perinatal loneliness and social justice

Most policies and interventions frame loneliness as an individual problem to be resolved by reducing isolation through increasing social connection.^57 80^ The perception that opportunities for increased social participation will lead to a reduction in loneliness is problematic because there are other factors involved.^81^ Loneliness is also driven by structural and sociocultural exclusion.^80^,^84^ Social policies addressing poverty, improving transport and investing in health and social care will also be key to preventing perinatal loneliness.^4553 57 80^,^84^

Specialist PMHS are commissioned to support people experiencing severe and, increasingly, moderate mental illness; the expansion of these services across the UK is hugely welcome.^85^ Despite investment in specialist PMHS, delivery has been patchy due to staff shortages.^86^ Further, people with mild to moderate conditions usually do not meet the threshold for this support. For people experiencing milder conditions, NHS Talking Therapies is often the default pathway, but often, these services do not have specialist knowledge to meet the specific needs of parents in the perinatal period. Investing in services to support these parents is imperative, as well as training general mental health professionals to identify and support people with perinatal mental illness. Other support might include increasing health visitor numbers, investing in perinatal social prescribing and expanding community-based support through Family Hubs and the voluntary sector.

Hearts & Minds Partnership (a UK voluntary sector perinatal mental health collaborative) and practitioners in our advisory group highlight the funding pressures experienced by service providers who deliver support for parents experiencing mild to moderate perinatal mental health conditions. Many services are closing due to short-term competitive funding cycles that lead to de-recommissioning despite excellent performance or national recognition. Cost-effective community-based services need to be developed in collaboration with voluntary organisations already successfully supporting underserved perinatal populations.

Some services support parents up to only 1 year post partum, but there is a growing understanding of the importance of continuing to support parents up to 2 or more years after birth, or even throughout the preschool period.^85^ Support for parents may drop off after the first year, and many parents return to work. These changes bring financial and emotional pressures for parents navigating the UK childcare system, which is non-universal and expensive, often costing parents more than their monthly housing cost.^87^ Moreover, flexible working options are limited. More choice and availability around parental leave may reduce loneliness by enabling parents to prioritise time or increase the amount of time to look after their own needs.^88^ In couples following the birth of a child, supporting shared care for both parents from birth may reduce the burden for one parent to take on all the caring responsibilities and potentially reduce exclusion.^21^

Sociocultural exclusion also contributes to loneliness. Parents for whom English is not their first language and parents who are refugees or migrants particularly struggle to hear about and engage with services.^53^ The heteronormative culture in services that are not inclusive of LGBTQ+ parents and/or fathers or co-parents also contributes to loneliness.^58^ Parents can feel excluded through a lack of inclusive language.^21^

## Recommendations for research and practice

In 2024, the Conservative UK Government stopped funding the Campaign to End Loneliness, which may be a signal that addressing loneliness will be deprioritised. It remains to be seen whether the current Labour government will reprioritise the issue, but early signs indicate their plan for the NHS is to focus on prevention. In this climate, the importance of preventing and reducing loneliness must be emphasised, with a focus on sociocultural and structural drivers in addition to individual support.

Adopting a social justice approach to addressing loneliness as outlined by Barreto and colleagues^57^ would enable societal changes to create a family-friendly culture in the UK that better supports parents and reduces feelings of loneliness. Changes to parental leave and childcare provision could be explored. Cuts to critical early-year services should be reversed, and infrastructure and transport could be improved to create child- and family-friendly cities. Future studies could explore the impacts of extended parental leave on loneliness through comparison to welfare systems in other countries. More inclusive practice is needed to overcome ageism, ableism, sexism, homophobia, heteronormativity, racism and related discrimination and microaggressions. Further research is required to explore how these sociocultural and structural factors can be addressed, perhaps through international comparative research, to understand different welfare structures and impacts on the experience of perinatal loneliness.

Future research will need to prioritise loneliness experienced by marginalised and vulnerable communities. Practitioners in our advisory groups identified groups whom they thought might be at additional risk, including younger and older mothers, care leavers, parents who have complex fertility journeys and/or have experienced previous losses and those who have children with health conditions and/or disabilities, including neonates. The growth in the diagnosis of neurodiversities including attention-deficit/hyperactivity disorder (ADHD) and autism, especially in women, is also worthy of increased attention as neurodiverse people are more likely to experience loneliness.^89^

New panel surveys should collect data on perinatal mental health and include measures of loneliness. This would enable increased understanding of prevalence and potential risk and protective factors.

Individual or group interventions to reduce loneliness should focus on parental mental health in addition to infant development. Interventions should be co-produced to maximise engagement and effectiveness. Interventions should be rigorously evaluated, including an exploration into who attends support and who is left behind, to understand if, how and why underserved communities are excluded from support.

## Limitations

This consensus statement contains the views of the researchers, professionals and people with lived experience who contributed to this paper. We recognise some perspectives may be absent. The perspectives of parents who adopt, and the services and professionals who support them, may not have been represented in this consensus statement. We invite reflection and discussion to expand on this statement and hope future iterations will represent more experiences.

## Data Availability

Data are available upon reasonable request.
