# Supplementary material for: Exploring perinatal loneliness as a key social determinant of perinatal mental ill health in the UK: findings from a multidisciplinary consensus statement exercise that mapped knowledge about measurement, prevalence, antecedents, impacts and interventions, and agreed future priorities for research, policy and practice
Source: BMJ Open. 2025 May 31;15(5):e085669. doi: 10.1136/bmjopen-2024-085669 (PMC12128407; doi:10.1136/bmjopen-2024-085669)
Supplement: Supplementary file 1 [file bmjopen-2024-085669.reviewer_comments.pdf]

PEER REVIEW HISTORY

BMJ Open publishes all reviews undertaken for accepted manuscripts. Reviewers are asked to complete a checklist review form and are provided with free text boxes to elaborate on their assessment. These free text comments are reproduced below.

ARTICLE DETAILS

Title (Provisional)

Exploring perinatal loneliness as a key social determinant of perinatal mental ill health in the UK. Findings from a multidisciplinary consensus statement exercise that mapped knowledge about measurement, prevalence, antecedents, impacts and interventions, and agreed future priorities for research, policy and practice.

Authors

Naughton-Doe, Ruth; Nowland, Rebecca; Kent-Marvick, Jacqueline; McKenna-Plumley, Phoebe; Lovett, Emily; Nguyen, Thuy-Vy; Hunter, Lindsay; Wallis, Nicola; Gaughan , Florence; Hall, Katie; Colella, Corinna; Wilson, Suzanne; Adlington, Katherine; Taylor Buck , Elizabeth; Shemery, Sarah; Finch, Naomi; Noone, Catrin

VERSION 1 - REVIEW

**Reviewer**	**1**
**Name**	**Folliard, Kelda**
**Affiliation**	**University of East Anglia**
**Date**	**13-Nov-2024**
**COI**	**None**

Thank you for developing this consensus piece which has scoped relevant literature to consider and comment on an important and under-investigated area of global perinatal social and mental health significance, with cross-disciplinary applicability. Please see below some points for consideration prior to resubmission.

Page 7, line 8: comment on the UK midwifery endorsement of perinatal mental health focus would be strengthened with reference to maternity transformation planning – e.g. Better Births and the Three-Year Delivery Plan for Maternity and Neonatal Services.

Methods: Was a recognised consensus development methodology used or drawn on? Are you able to explain the decision behind your chosen approach to literature scoping and evaluation? Is there an example of the spreadsheet used which summarises the included papers and how these were mapped with key information? It would be interesting to understand more about what is meant by practice insights and how this informed the group’s thinking. Were all the authors working independently and then sharing findings?

What was the process of discussing with the advisory group? Could you provide any of this information as supplementary material?

Page 13, line 48: typo sentence ‘which in Local Authorities to deliver’

Page 13, line 51: consider whether you wish to interrogate this further – for example implications of this funding being short term and that ‘Family Hub’ suggests one accessible physical space when actually provision of physical space is more limited with geographical variation in service models (anecdotally). This may be relevant when considering loneliness. Although I appreciate the scheme has not been formally evaluated yet.

Page 14, line 45: Antenatal classes – may be worth also commenting on the lack of consistency in delivery and move away from traditional in-person class approach, may have impacted the ability to form social connections.

Page 15, line 23: typo – ‘the scoping review found only study’

Page 16, line 40: comment on perinatal mental health specialist services and people with mild to moderate condition not meeting threshold for support. Needs rethinking as specialist services are not targeted for mild to moderate presentation. IAPT/Talking Therapies services are for mild to moderate presentations and may offer opportunities for peer engagement. Although it is still questionable whether services as a whole effectively address loneliness, this just needs clarifying to show that differentiation between service remits is understood.

Page 18, line 25: typo ‘neonatal babies’ – neonates or babies?

VERSION 1 - AUTHOR RESPONSE

**Feedback from peer review and editors**	**Our response/changes we’ve made**
Page 16, line 40: comment on perinatal mental health specialist services and people with mild to moderate condition not meeting threshold for support. Needs rethinking as specialist services are not targeted for mild to moderate presentation. IAPT/Talking Therapies services are for mild to moderate presentations and may offer opportunities for peer engagement. Although it is still questionable whether services as a whole effectively address loneliness, this just needs clarifying to show that differentiation between service remits is understood.	Thank you for highlighting this. We have expanded this section to make the distinction between different types of services clearer, and to explain which services need to be expanded.<br><br>*‘Specialist perinatal mental health services are*

	commissioned to support people experiencing severe, and, increasingly, moderate mental illness; the expansion of these services across the UK is hugely welcome [85]. Despite investment in specialist perinatal mental health services, delivery has been patchy due to staff shortages [86]. Further, people with mild to moderate conditions usually do not meet the threshold for this support. For people experiencing milder conditions, NHS Talking Therapies is often the default pathway, but these services do not usually have specialist knowledge to meet the specific needs of parents in the perinatal period. Investing in services to prevent perinatal mental ill health arising, and to support those with mild-moderate needs, is imperative. This includes expanding health visitors, midwives and community-based support through Family Hubs and the voluntary and charitable sector. In the UK, these services are often delivered

	by voluntary sector providers.'
Page 7, line 8: comment on the UK midwifery endorsement of perinatal mental health focus would be strengthened with reference to maternity transformation planning – e.g. Better Births and the Three-Year Delivery Plan for Maternity and Neonatal Services.	We have added reference to these important documents- thank you.<br><br>The National Maternity Review: Better Births (2016) [12] and the NHS England 'Three-year delivery plan for maternity and neonatal services' [13] emphasise the importance of access to perinatal mental health support.
Page 13, line 48: typo sentence 'which in Local Authorities to deliver'<br><br>Page 15, line 23: typo – 'the scoping review found only study'<br><br>Page 18, line 25: typo 'neonatal babies' – neonates or babies?	We have made these changes in the document
Page 13, line 51: consider whether you wish to interrogate this further – for example implications of this funding being short term and that 'Family Hub' suggests one accessible physical space when actually provision of physical space is more limited with geographical variation in service models (anecdotally). This may be relevant when considering loneliness. Although I appreciate the scheme has not been formally evaluated yet.	We agree this section needed to be expanded. We have added a couple of sentences to note the access and funding challenge and the differences in delivery models (with references).<br><br>"Specialist perinatal mental health services are commissioned to support people experiencing severe, and, increasingly, moderate mental illness; the expansion of these services across the UK is hugely welcome [85]. Despite investment in specialist perinatal mental health services, delivery has been patchy due to staff shortages [86]. Further, people with mild to moderate conditions usually do not meet the threshold for

	this support. For people experiencing milder conditions, NHS Talking Therapies is often the default pathway, but often these services do not have specialist knowledge to meet the specific needs of parents in the perinatal period. Investing in services to support these parents is imperative as well as training general mental health professionals to identity and support people with perinatal mental illness. Other support might include increasing health visitor numbers, investing in perinatal social prescribing, and expanding community-based support through Family Hubs and the voluntary sector.”
Methods: Was a recognised consensus development methodology used or drawn on? Are you able to explain the decision behind your chosen approach to literature scoping and evaluation? Is there an example of the spreadsheet used which summarises the included papers and how these were mapped with key information? It would be interesting to understand more about what is meant by practice insights and how this informed the group’s thinking. Were all the authors working independently and then sharing findings? What was the process of discussing with the advisory group? Could you provide any of this information as supplementary material?	We have expanded the methods section to include more details of the consensus meeting approach we used, including adding references. We have also added more detail on lived experience and professional advisory group involvement in the Patient and Public Involvement Statement.<br><br>We have addressed a limitation of our research in the limitations section- we did not use a method such as Delphi, but a consensus meeting approach which is appropriate in the context of

	limited time and resources. However, the consensus meeting approach is not as rigorous as the Delphi method (or similar) so we have explained the limitations clearly.<br><br>We cannot share the table we developed as it was intended as a tool for discussions and is in note-form rather than public-facing text.<br><br>'*The Parental Loneliness Research Group developed a consensus statement through a series of online meetings between October 2023 and May 2024. We followed a consensus meeting approach [19] previously utilised to develop a similar statement for older adults [20]. The first meeting was attended by academics and practitioners in the UK and USA who had attended the 1st Perinatal Loneliness Annual Conference in October 2024. It included those working in Social Policy, Sociology, Social Work, Psychology, Midwifery and Psychiatry, a Parent-Infant Psychotherapist, and people delivering perinatal creative and cultural interventions. We identified and invited other experts to subsequent meetings including the authors of any literature reviews on perinatal loneliness.*<br><br>*The lead author took notes*

	at each meeting and produced a list of points to consider based on Prohaska's approach [20]. These were: research methods used in perinatal loneliness research; conceptual definitions; measurement and prevalence of perinatal loneliness; experiences, influences, and causes of perinatal loneliness; individual, structural, and cultural drivers and risk factors; impacts of loneliness; barriers to overcoming loneliness; interventions for overcoming loneliness; and intervention mechanisms. Authors RND and RN developed a spreadsheet of the above items for co-authors to map key information from their published and unpublished research. Practitioners in the group completed a spreadsheet to reflect on their experiences of supporting parents to supplement insights from published papers. We then used the contents of the spreadsheets to structure our online discussions. Through a process of co-writing on a shared MS Google Document and discussions in our meetings, we agreed on a consensus statement. A summary of our conclusions is presented below.'
Page 14, line 45: Antenatal classes – may be worth also commenting on the lack of consistency in delivery and move away from traditional in-person class approach, may have impacted the ability to form social connections.	We have added a sentence to address this:<br><br>'However, there are issues around equitable access to such programmes, consistency of delivery, and the impact of transitioning classes to online platforms

	on social connectedness.’
Author changes	We have added some changes to papers that have now been published and proof-read.<br><br>We added a section on volunteering as an intervention after consulting with the advisory group – this in the interventions section.<br><br>We have also added a lack of reference to adoption specifically as a limitation.<br><br>We have thoroughly proofread the document and made some changes to improve reading quality.
